# Towards improving the PF1B beamline *McStas* model through simulations of a beam characterization experiment

**DOI:** 10.1107/S1600576726001639

**Published:** 2026-03-28

**Authors:** Clément Desalme, Jason Pioquinto, Valentin Czamler, Stefan Baeßler, Kazimierz Bodek, Valery Nesvizhevsky, Dagmara Rozpędzik, Katharina Schreiner, Torsten Soldner

**Affiliations:** a Institut Laue-Langevin, CS 20156, 38042 Grenoble Cedex 9, France; b Doctoral School of Exact and Natural Sciences Jagiellonian University, 30-348 Cracow Poland; c Marian Smoluchowski Institute of Physics Jagiellonian University, 30-348 Cracow Poland; d Department of Physics, University of Virginia, Charlottesville, VA 22904-4714, USA; e Université Grenoble Alpes, CNRS, Grenoble INP, LPSC-IN2P3, 38026 Grenoble, France; f Laboratoire Kastler Brossel, UPMC-Sorbonne, CNRS, ENS-PSL, Collège de France, 75005 Paris, France; g Vienna Doctoral School in Physics, Faculty of Physics, University of Vienna, 1090 Vienna, Austria; h Marietta Blau Institut Austrian Academy of Sciences, 1010 Vienna Austria; Technical University of Denmark, Denmark

**Keywords:** *McStas* simulations, time of flight, cold neutron beams, instrument models

## Abstract

The PF1B instrument at the Institut Laue–Langevin (ILL) delivers a high-flux cold neutron beam through the H113 neutron guide. In the years following the characterization published with the highest polarization in 2006, radiation-damaged sections of the guide have been replaced and the in-pile part has been upgraded. In 2024, a new experiment using time of flight was performed to characterize the instrument. Simulations intended to reproduce this experiment have been performed with *McStas* using an already existing model of H113. In this work, we present a first comparison of experimental and simulated time-of-flight spectra, showing overall good agreement but discrepancies which might be reduced by adjusting the existing *McStas* model. Parameter optimization is ongoing, aiming at a more accurate description that would serve for future experiments on PF1B.

## Abstract 2

*McStas* simulations of a recent time-of-flight characterization of the PF1B instrument at the Institut Laue–Langevin show good agreement with the current beamline model, while also highlighting opportunities to refine some parameters of the simulation using the collected time-of-flight data as a benchmark.

## 1. Introduction

The PF1B instrument is part of the versatile suite at the Institut Laue–Langevin (ILL) in Grenoble. This instrument is mainly used for nuclear or particle physics experiments, *e.g.* to study nuclear structure via γ-spectroscopy (Jentschel *et al.*, 2017 ▸), ternary fission (Jesinger *et al.*, 2002 ▸; Gagarski *et al.*, 2016 ▸) or the nuclear weak interaction (Vesna *et al.*, 2005 ▸; Vesna *et al.*, 2008 ▸), to search for the neutron electric charge (Persoz, 2024 ▸) or the neutron electric dipole moment (Fedorov *et al.*, 2010 ▸; Chanel, 2021 ▸; Schulthess *et al.*, 2022 ▸), or to measure correlation coefficients in free neutron β-decay (Soldner *et al.*, 2004 ▸; Mund *et al.*, 2013 ▸; Märkisch *et al.*, 2019 ▸; Beck *et al.*, 2020 ▸). The H113 neutron guide delivers cold neutrons that are moderated in the vertical cold source (VCS) located in the ILL reactor’s heavy water moderator tank and which is composed of liquid and gaseous deuterium maintained at a temperature of 20 K (Ageron, 1989 ▸). At the guide exit, more than 70 m away from the reactor pool, the cold neutron capture flux is 2.2 × 10^10^ n cm^−2^ s^−1^ (Petoukhov *et al.*, 2023 ▸) over a beam area of 6 × 20 cm (Häse *et al.*, 2002 ▸). A beam-averaged polarization of 99.7% can be reached at the instrument, using a V-bender supermirror polarizer (Petukhov *et al.*, 2016 ▸; Petukhov *et al.*, 2019 ▸; Petoukhov *et al.*, 2023 ▸), allowing for studies using polarized neutrons (Bodek *et al.*, 2019 ▸).

The PF1B instrument was commissioned in August 1999, succeeding the previous PF1 cold neutron beamline that was located at another beamport in ILL’s second experimental hall. It delivers a more intense flux than its predecessor thanks to the *m* = 2 H113 guide, which is the first ballistic neutron guide that was developed at S-DH in Heidelberg (Häse *et al.*, 2002 ▸). The instrument was initially characterized via time of flight (TOF) (Abele *et al.*, 2006 ▸), and regular gold foil activation measurements have also been performed at least once a year ever since its commissioning. After a few years of operation, before the characterization measurements (Abele *et al.*, 2006 ▸), a decrease in neutron flux was observed. A visual inspection of the guide sections showed that the Borofloat supermirror coating of the guides had been altered over time due to the high fluence encountered near the reactor pool. Since several neutron guide sections suffered from irreversible radiation damage, they were replaced by supermirrors in Borkron, while the initial section was upgraded using supermirrors in float glass (Boffy *et al.*, 2012 ▸; Boffy, 2016 ▸). Measurements revealed afterwards that the flux at the guide exit surpassed that measured after PF1B’s commissioning, as expected from the initial section upgrade (Abele *et al.*, 2006 ▸).

In order to keep an up-to-date status of the beamline, a new experimental campaign was undertaken in 2024 to reassess the beamline properties and complement the flux monitoring data. These measurements, described in Section 2, were accompanied by *McStas* ray-tracing simulations (Section 3), showing promising agreement with the experimental data.

## 2. PF1B characterization experiment

### 2.1. Experimental setup

The objective of the beam characterization carried out on PF1B was to obtain TOF spectra of the cold neutron beam at different positions across the exit of the H113 guide, also recording the spectrum as a function of the angle relative to the beam axis (Pioquinto *et al.*, 2024 ▸).

**Figure 1:**
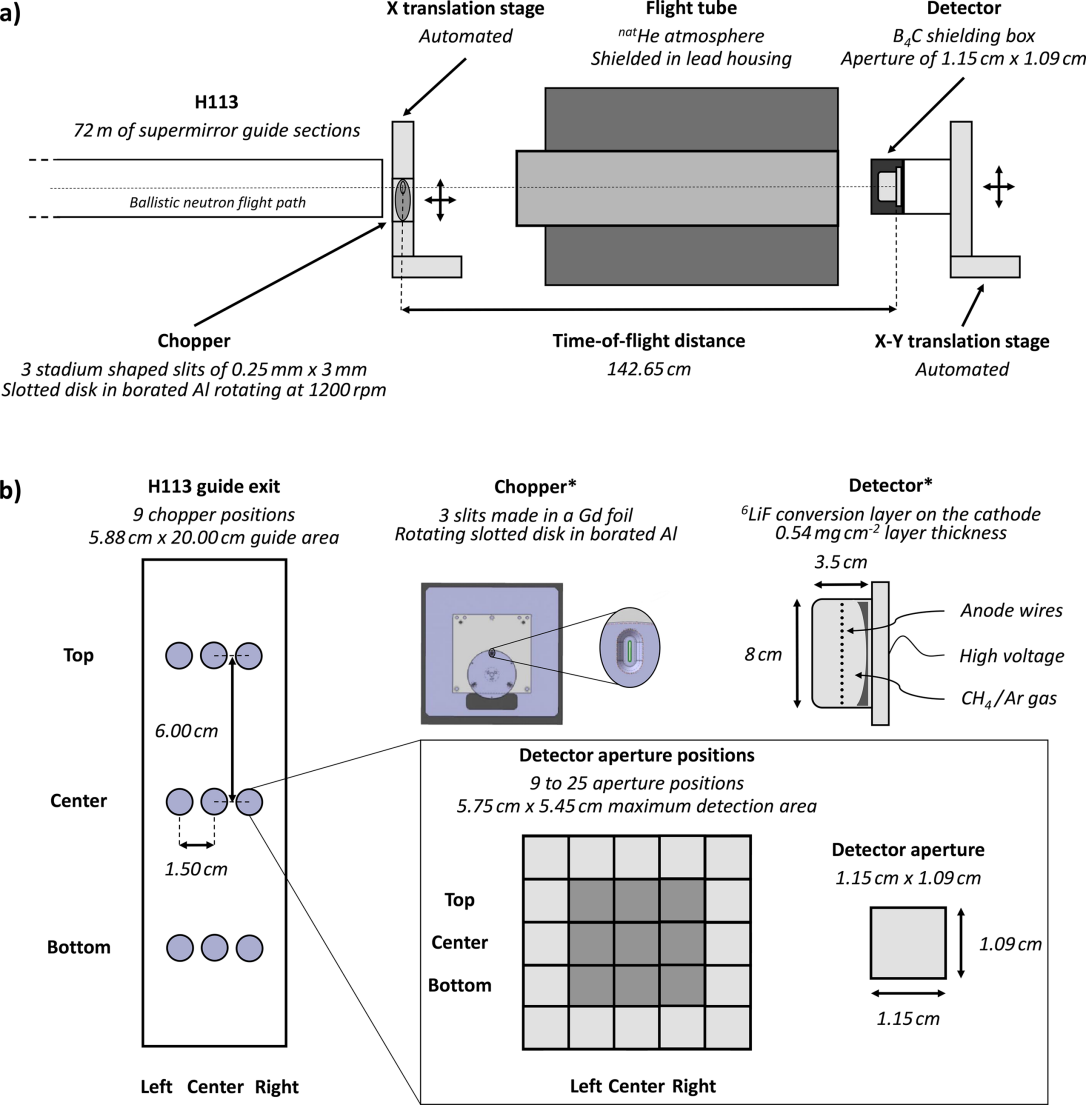
(*a*) Experimental setup used during the characterization experiment of PF1B (Pioquinto *et al.*, 2024 ▸). (*b*) Descriptions of the chopper and the detector, as well as the performed measurement scheme showing the chopper and detector aperture positions. The detector is seen from the reactor side in the direction of neutron flight. The chopper and detector diagrams marked with an asterisk (*) are adapted with permission from Roulier *et al.* (2019 ▸) under a Creative Commons Attribution 4.0 International License, https://creativecommons.org/licenses/by/4.0/.

The experimental setup is schematically illustrated in Fig. 1 ▸(*a*), and details of the neutron guide geometry can be found in the reports by Häse *et al.* (2002 ▸) and Abele *et al.* (2006 ▸). Neutrons first emerge from the 0.3 mm thick magnesium window at the guide exit before passing through a chopper (Roulier *et al.*, 2019 ▸) that is mounted on a motorized translation stage and a manual elevation stage. The chopper consists of a rotating disk of 62 mm diameter in borated aluminium with a gadolinium foil glued on top of it. Both the disk and the foil feature three evenly spaced stadium-shaped slits, each of *l* = 0.25 mm wide and *L* = 3 mm high, positioned at *r* = 26 mm from the rotation axis as measured from their inner edge. An aluminium holder, in which a fixed slit of the same shape is machined, defines the neutron pulses when one of the rotating slits aligns with it. The disk rotation frequency was set to 1200 rpm during the experiment, *i.e.* to *f* = 20 Hz. Accounting for the three slits, this translates in a TOF pulse frequency of 60 Hz, *i.e.* a frame duration of *t*_frame_ = 16.67 ms. The TOF relation links the neutron velocity, which can be converted to the neutron wavelength λ using the de Broglie formula involving the neutron mass *m*_n_ and the Planck constant *h*, with the TOF distance *d* and the time of flight *t*,
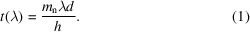
The maximum wavelength λ_max_ which can be detected by the chopper without frame overlap is given for *t*(λ_max_) = *t*_frame_. For a TOF distance of *d* = 142.65 cm [Fig. 1 ▸(*a*)], we obtain λ_max_ = 46.23 Å.

The stadium-shaped slits can be approximated by rectangular slits of the same dimensions. The angular velocity of the chopper is ω = 2π*f* and the tangential velocity of the slits is *v* = ω*r*. The opening function of a rectangular slit in a chopper, written *T*(*t*), is given by a triangular function which is symmetric with respect to the base time *t*_0_ = *l*/2*v*. This function can be normalized to unity. 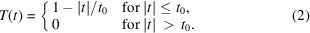
The FWHM Δ*t*_FWHM_ of this function is such that Δ*t*_FWHM_ = *t*_0_. The wavelength resolution of the TOF setup is limited by the chopper opening function, 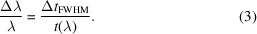
The slow rotational velocity of the chopper disk means that the short-wavelength part of the TOF spectrum is more dominated by the timing width that is produced by the chopper, 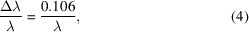
where λ is in ångströms.

Downstream of the chopper, neutrons travel through a 99 cm long flight tube located 40 cm away from the rotating disk. The tube is covered on the inside with 0.55 cm thick boron-loaded rubber and closed at the ends with thin aluminium foil, while being continuously flushed with natural helium to minimize air scattering and absorption on the way to the detector. It is enclosed in a lead housing for γ shielding purposes.

A neutron detector (Roulier *et al.*, 2019 ▸), placed 5 cm after the flight tube, is enclosed in a shielding box made of boron-loaded rubber of 0.55 cm thickness (with two layers on the front panel, *i.e.* 1.1 cm shielding thickness). On the front, an aperture of 1.15 cm in width and 1.09 cm in height is cut in the shielding [Fig. 1 ▸(*b*)]. The detector, in its shielding box, is mounted on an automated translation–elevation stage, which allows precise scanning of the beam profile in two dimensions.

### 2.2. Measurement scheme

The experiment’s primary goal was to obtain TOF spectra at several guide exit and detector positions in order to quantify the beam divergence and the neutron spectrum at different positions and angles. As depicted in Fig. 1 ▸(*b*), measurements on the beamline were performed by first adjusting the placement of the chopper relative to the H113 guide exit covering nine different positions. For each chopper position, a 3 × 3 scan over the aperture was performed, and at the (Top, Center) and (Center, Center) positions the scan density was increased to 5 × 5. An aperture-sized scan means that the step sizes in the *X* and *Y* directions were set equal to the corresponding dimensions of the detector aperture. This measurement scheme makes sure that the TOF spectra cover the scan range at the detector position without overlap or unsampled regions.

In addition, a measurement was made to determine the brightness of the VCS, denoted as *B*:
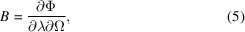
which is defined with the neutron flux Φ in cm^−2^ s^−1^, the neutron wavelength λ in ångströms and the solid angle of neutron emission Ω in steradians. The narrow collimation by both the chopper and the detector aperture accepts only neutrons that have undergone one reflection in the guide, therefore minimizing effects from the supermirror on the measured spectrum and allowing us to access the VCS brightness. A detector scan was performed at the (Center, Center) chopper position, with an additional pinhole aperture of 2 mm diameter made in a cadmium sheet that was placed on top of the detector aperture. Simulations of this measurement are very time consuming because of the small acceptance of the setup and the model of the instrument in *McStas*. Further results will be presented in a future publication.

## 3. *McStas* simulations

### 3.1. Experiment model

A simulation model of H113 was developed in *McStas* (Lefmann & Nielsen, 1999 ▸; Willendrup & Lefmann, 2020 ▸; Willendrup & Lefmann, 2021 ▸) by PF1B’s instrument scientists in the years following the replacement of the initial guide section. It is based on an internal technical document (Boffy *et al.*, 2012 ▸) precisely describing the guide system over 85 m, from the VCS in the pool up to the final beamstop in the experimental area. This model accounts for the particular geometry of the ballistic guide, divergent in the first few metres and convergent in the last few, using only Guide_gravity() components. It also takes into consideration aluminium vacuum windows in guide section transitions or in guide interruptions due to the presence of safety shutters or fast valves.

The model of the experiment includes relevant components from the guide exit up to the lithium fluoride detector conversion layer (Fig. 1 ▸). The chopper system has been implemented not as a moving slotted disk but as nine different rectangular slits of the same width and height as the stadium-shaped ones, placed at the nine different chopper positions taken during the experimental campaign [Fig. 1 ▸(*b*)]. Each detector pixel was simulated using a Monitor_nD() detector that was parameterized to record a TOF distribution in the wavelength range that had been measured during the characterization experiment. In order to optimize the com­putation time and be able to simulate several slits in parallel, neutrons were propagated once from the cold source to the exit of the nine slits and saved at this position in a Monte Carlo Particle List (MCPL) file (Kittelmann *et al.*, 2017 ▸). This file, comprising 5669362482 probability packets, was run once as an input to simulate the propagation through the rest of the experiment up to the detector, with an absorbing mask to select only neutrons coming from the slit of interest. In the simulation, a perfect vacuum was assumed as a first approximation in H113 and from the guide exit to the detector. Gravity was also not included since it increased the computation time by a factor of ten.

### 3.2. Simulation results

**Figure 2:**
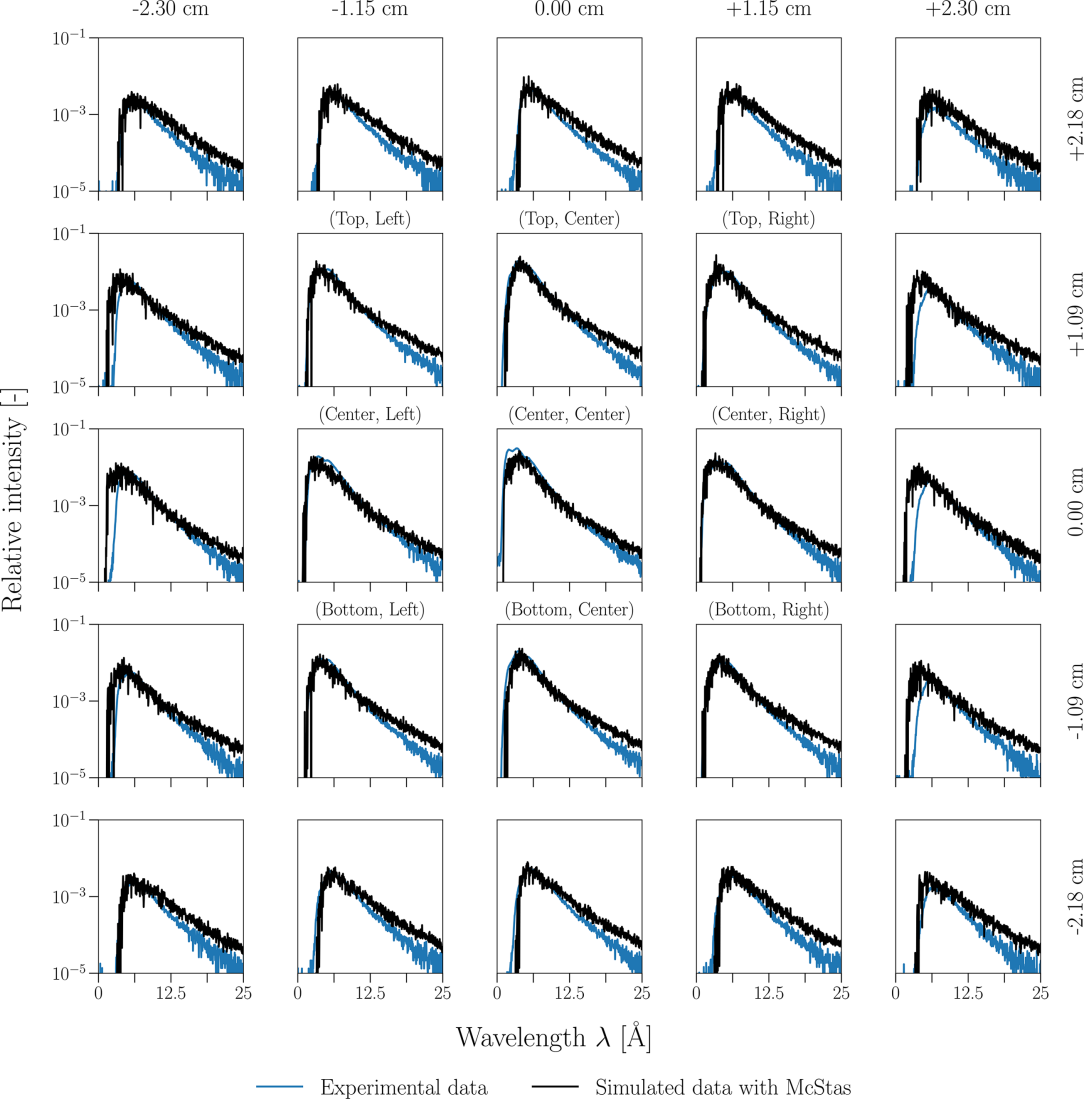
Experimental and simulated TOF spectra at the (Center, Center) chopper position showing the 25 detector aperture positions, therefore covering a total detector area of 5.75 × 5.45 cm.

Experimental results from the characterization experiment were used as a benchmark and compared with the simulated data from *McStas* at the (Center, Center) position (Fig. 2 ▸), which is one of the nine chopper positions where data were taken. The figure compares TOF spectra of experimental and simulated data, both of which have been normalized by the respective integrated count rate over the 25 pixels. The wavelength dependency of the detector efficiency is also taken into account (Roulier *et al.*, 2019 ▸). The simulated spectra across the detector aperture positions of the 5 × 5 grid exhibit consistency in both shape and intensity distribution regarding the corresponding experimental data. Each pixel’s spectrum in the simulation shows the expected characteristic shape measured in the last extensive characterization experiments (Abele *et al.*, 2006 ▸). For each, there is a rapid rise at short wavelengths, with a well defined peak around 3–6 Å depending on the angle to the beam axis, and a long gradually decaying tail extending toward 40–45 Å (only wavelengths up to 25 Å are represented for clarity and ease of data visualization).

One can observe some spatial variations across the grid, as the central pixels tend to show slightly higher intensity than the peripheral ones, which is expected since the peripheral pixels mainly collect neutrons coming from the beam divergence. The spectra of the central pixels also tend to peak at a lower wavelength. This is again expected since the outer pixels correspond to larger angles to the beam axis and thus to the guide mirrors, resulting in higher wavelengths. One interesting effect that is yet to be understood is the overestimation of neutrons above 15 Å, which is more significant on the side columns than in the rest of the detector aperture positions.

Overall, the comparison demonstrates that the *McStas* model provides a satisfying representation of the cold neutron beam of PF1B, capturing the essential physical processes, and thus serves as a reliable basis for further improvements and predictive studies later on.

### 3.3. Simulation improvement

In order to strengthen the agreement between experimental and simulated data, several improvements are underway to represent the experiment better in *McStas*.

Besides the implementation of gravity during the propagation of neutrons in H113, some of the most important model parameters to be validated or adjusted are those of the cold source spectrum definition in *McStas*. The current model of the VCS is based on previous measurements and simulation work conducted by several ILL scientists over the years (Andersen, 2014 ▸; Farhi, 2015 ▸). With the data from the pinhole measurement at the (Center, Center) chopper position that were measured in addition to the TOF studies during the characterization experiment, the spectrum of the cold source can be verified and possibly adjusted more reliably than from the measurements with a larger aperture, as explained previously (Abele *et al.*, 2006 ▸). With the validated model of the cold source spectrum, one can then tune the different supermirror coating parameters, such as the *m* value or the *R*_0_ reflectivity parameter, or the waviness of the supermirrors.

Minor corrections to the TOF distance value, as well as to the positions of components in H113, or in the experiment, could also be considered, accounting for possible misalignment or geometric imperfections. The final iterations could also implement an ambient air atmosphere after the guide exit, and a natural helium atmosphere in the flight tube, in order to account for scattering and absorption over the entire flight path of the neutrons. Another improvement could consist of replacing the Al_window() component, currently used to model the aluminium windows in the beamline or the experiment, by a component that takes into account both absorption and scattering in aluminium. Lastly, the boron-loaded rubber shielding could also be defined as a custom material taking into account both scattering and absorption cross sections, instead of a total absorber as done with the Absorber() component.

## 4. Conclusion

The 2024 characterization campaign of the PF1B instrument has enabled a reassessment of the H113 neutron guide feeding PF1B. Data taken during the experiment are now being used as a benchmark to refine the *McStas* model of the beamline. A validated and accurate representation will support more precise data analysis for future experiments and allow the identification of problematic guide sections in H113. It lays the groundwork for incorporating additional available optical elements of the instrument into the simulation framework, such as the new solid-state supermirror polarizer (Petoukhov *et al.*, 2023 ▸).

## Supplementary Material

Characterization data of the PF1B instrument during summer 2024, realized under experiment 3-07-420.: https://doi.org/10.5291/ILL-DATA.3-07-420

## Data Availability

Simulation data will be made available upon request. The experimental data follow the ILL data policy and can be made available upon request.
